# Does occupational noise matter amongst manufacturing (small and medium enterprises) workers? Empirical evidence from Magaba, Mbare, Zimbabwe

**DOI:** 10.4102/sajcd.v67i2.680

**Published:** 2020-03-03

**Authors:** Miston Mapuranga, Eugine T. Maziriri, Ralebitso K. Letshaba, Anos Chitamba

**Affiliations:** 1 School of Managerial Leadership, The Da Vinci Institute for Technology Management, Johannesburg, South Africa; 2 Department of Business Management, Faculty of Economic and Management Sciences, University of the Free State, Bloemfontein, South Africa; 3 Department of Human Resource Management, Faculty of Management Sciences, Vaal University of Technology, Vanderbijlpark, South Africa; 4 Graduate School of Business and Leadership, College of Law and Management Studies, University of KwaZulu-Natal, Durban, South Africa

**Keywords:** attitudes towards occupational noise exposure, job performance, occupational noise, perceived susceptibility to hearing loss, SME workers

## Abstract

**Background:**

The significance of how occupational noise can influence attitudes towards occupational noise exposure, susceptibility to hearing loss and job performance has generally been neglected in the past studies.

**Objectives:**

The aim of this study was to determine the impact of occupational noise on attitudes towards occupational noise exposure, susceptibility to hearing loss and job performance of manufacturing small and medium enterprises (SMEs) workers in Zimbabwe.

**Method:**

A survey was conducted involving 250 respondents, including manufacturing SME workers, and the hypotheses were analysed by applying structural equation modelling.

**Results:**

Occupational noise had a positive and significant effect on attitudes towards occupational noise exposure and perceived susceptibility to hearing loss amongst manufacturing SME workers. In addition, attitudes towards exposure to occupational noise and the perceived susceptibility of hearing loss have had a positive and significant impact on manufacturing SME workers’ job performance.

**Conclusion:**

The novelty of the research is its analysis of occupational noise as an indicator of attitudes towards occupational noise exposure and susceptibility to hearing loss as well as job performance. This study provides practitioners with beneficial implications. Collective knowledge on occupational noise could help manufacturing SME managers in recognising the perceptions of employees on occupational noise and how it ultimately affects job performance. Moreover, this study is intended to add new knowledge to the current body of African occupational noise literature – a context that has not received much research attention in developing countries.

## Introduction

Globally, noise has become a typical work-related danger and is probably the biggest reason for hearing loss (Nelson, Nelson, Concha-Barrientos, & Fingerhut, 2005). Occupational hearing loss is undeniably an inevitable condition of occupational health taking place all over the world. It could be exacerbated by a couple of components at workplace, for instance, ototoxic substances and noise (Liu et al., 2015). A basic medical concern with economic outcomes is noise-induced hearing loss (NIHL) at workplace (Lie et al., 2016). Occupational noise-induced hearing loss (ONIHL) is a disease that arises from exposure to noise in working environment or from non-noise agents (Martínez, 2012). It takes place at a wide range of workplaces, including printing, food production, metal mining and assembly (Liu et al., 2015). In a low- and middle-income (LAMI) nation such as Zimbabwe, the *Factories and Works Act*, Chapter 14:08 (Zimbabwe Government, 1996a) anticipates that supervisors would lawfully make every single viable move for the welfare and protection of employees and individuals at their premises. This Act specifies that all workers ought to be appropriately trained and, when under supervision, are appropriately supervised by a capable individual, furnished with personal protective equipment and clothing where necessary, and that these are utilized, so that these workers are shielded against risky machinery, dangerous procedures, as well as dust and noise (Chadambuka, Mususa & Muteti, 2013).

Singh, Bhardwaj and Deepak (2013) attest that exposure to occupational noise (ON) actually leads to ONIHL. Obviously, ONIHL has become an unavoidable work-related condition throughout the world, and is accounted to occur in quite a number of workplaces. As commented by Dudu and Jeche (2014):

Approximately 11 million people in the United States alone are exposed to hazardous noise levels in the workplace and 9 per cent of the total workforce in Sweden is exposed to this conceivably dangerous level of noise. (p. 22)

In China, ONIHL is predominantly found ‘among workers in municipal solid waste landfills’ (Liu et al., 2015:1). A large portion of these examinations are directed in developed nations. Evidence from LAMI countries such as India shows that employees in Indian SMEs perform in a less technically developed and much noisier environment, and yet very limited literature is available on ONIHL in such contexts (Singh et al. 2013).

Arising from the previously mentioned international investigations, research focussing on ON in manufacturing SMEs, especially in Africa, is inadequate. Al-Arja and Awadallah (2020) have clarified that in developed nations, not many investigations have examined ON. Therefore, it would be naïve to assume a priori that findings from developed countries in Europe or from the United States of America, or even from the newly developed countries in Asia, apply in Africa. Perhaps, an exploration on the effect of ON on attitudes towards ON exposure, susceptibility to hearing loss and job performance in the African settings could yield insightful outcomes in comparison to those from the developed nations of the world. Subsequently, neither to affirm nor disconfirm past investigations, this investigation focuses on ON in manufacturing SMEs of Africa. This was long overdue, as this lacuna merits exact assessment on account of an overlooked setting of manufacturing SMEs in developing nations.

Even though past investigations have examined the impact of ON on human well-being because of unidentified reasons, small enterprises, industries and workshops have not been included, although limited studies, especially in LAMI nations, have directed their attention towards this issue (Jabbari et al. 2016). Anjorin, Jemiluyi and Akintayo (2015) observe that research has been conducted to assess industrial noise in the refining, mining, oil and gas, construction and manufacturing industries, and the findings have shown that a high percentage of industrial workers in these industries have been subjected to more than 85 decibels (A-weighted) (dB[A]) noise levels. Despite this evidence, high noise levels have been underestimated in small enterprises in LAMI nations, such as Zimbabwe – focus of this article. To date, few studies have investigated occupational hazards, such as noise levels in the working environment, in Zimbabwe. This research was conducted in a different context, focusing on the following industries in Zimbabwe: prevalence of NIHL amongst mining employees (Chadambuka et al., 2013); occupational health and safety concerns amongst workers in the wood processing industry in Mutare (Jerie, 2012); study of hearing protection system (HCP) at a mining company (Mutara & Mutanana 2015) and evaluation of industrial noise levels in plastics industry (Dudu & Jeche, 2014).

In this way, deducing from the previously mentioned examinations directed on Zimbabwe, it is noted that studies concentrating on how ON affects attitudes towards ON exposure, susceptibility to hearing loss and job performance amongst manufacturing SME workers is regularly portrayed as shallow and needs further scholarly introspection. Moreover, lack of research on ON, attitudes towards ON exposure, susceptibility to hearing loss and job performance relationship are without doubt incomprehensible and warrant scholarly examination. Maybe the most convincing contention for examining the effect of ON on attitudes towards ON exposure, susceptibility to hearing loss and job performance emanates from the way the past investigations (Girard et al., 2015, p. 88; McTague et al., 2013, p. 3; Sriopas, Chapman, Sutammasa, & Siriwong, 2016, p. 1) on these constructs have, for the most part, centred around large firms and little was examined in the case of SMEs. This is all the more astonishing, considering that SMEs are commonly seen as the engine for business growth, financial progress and transformation in both developing and developed nations (Chinomona & Pretorius, 2011). Besides, these earlier investigations have, to a great extent, focused on developed nations (Chinomona, Lin, Wang, & Cheng, 2010). In this way, little is known on these issues in SAMI nations of the world, for example, African nations – specifically Zimbabwe. Therefore, this lacuna merits observational assessment on account of the disregarded setting of manufacturing SMEs in developing nations.

Furthermore, it is important to point out that not many scholars (if any) have used structural equation modelling (SEM) to check the causal links of ON, attitudes towards ON exposure, susceptibility to hearing loss and job performance. Regarding this study’s conceptual model, it is exceptional as it is tested in a developing nation setting.

This article pursues a structured set. To begin with, this examination is placed in context. Secondly, the empirical literature for the study’s variables is reviewed, thus prompting development of research hypotheses. Thirdly, the section on research design and methodology follows. Finally, there is a presentation of findings, discussions, conclusion, limitations and directions for the future research.

## Contextualisation of the study

### Background of the study area

This section provides details about the study site, Magaba, in Mbare, Harare. Mbare township is a high-density suburb, originally designed as a dormitory location to house black Africans working as domestic servants and industrial workers in Harare city (Helliker, Chiweshe, & Bhatasara, 2018; Zinyemba & Changamire, 2014). Magaba (meaning ‘empty tins’), a geographical area within Mbare, is approximately 3.9 km from Harare’s central business district (Helliker et al., 2018). It was unofficially established in the 1950s by a small group of tinsmiths who produced tin cans and other steel products to generate income. Magaba is also home to businessmen running informal businesses ranging from welding, tinkering and engine mechanics to sales and catering of building materials (Zinyemba & Changamire, 2014). In addition, heavy-duty businesses, requiring technical skills as well as physical power, characteristics mainly attributed to men in Zimbabwe’s society, are primarily established in Magaba (Zinyemba & Changamire, 2014). Dongo (2016) discovered that in Magaba’s residential-cum-industrial area, the sound of welding machines could be deafening; to communicate, and to be audible, people usually shout over the noise.

### Rationale and importance of selecting manufacturing small and medium enterprises and their workers

Laird, Olsen, Harris, Legg, and Perry (2011, p. 145) note that SMEs face unique problems in the area of occupational health and safety (OH&S) as opposed to larger companies, primarily because the risk of occupational hazards is greater and the capacity to mitigate hazards is lower in SMEs. Similarly, Reinhold, Järvis and Tint (2015) claim that ON concerns in small- and medium-sized businesses are increasingly an after-effect of inadequate hazard management and lack of resources than the real size of present hazards. Small businesses are widely acknowledged to be exposed to extreme ON and have limited resources to monitor such risk, but the literature, in general, tends to focus on small businesses as a matter of regulatory and enforcement (Hasle, Limborg, Kallehave, Klitgaard, & Andersen, 2012). Employees of SMEs are subjected to industrial noise from a wide range of sources, such as compressors and hydraulic machines in garages, warehouses and repair areas, portable power tools, heavy machinery and other equipment (Anjorin et al., 2015). Moreover, NIHL has been recorded in the five main occupational illnesses in Zimbabwe (Chadambuka et al., 2013).

## Empirical literature

This section centres on exploring literature on the variables under scrutiny.

### Occupational noise

Abulude, Fagbayide and Akinnnusotu (2018) state that noise should be seen as a performance impediment within an organisation. Wang, Qin, Lui, Han and Chen (2013) elucidate workplace noise as a repetitive sound heard at a place of work. Occupational noise is defined in a similar vein as any unwanted sound being produced in working environments (Al-Arja & Awadallah, 2020). Zare et al. (2015) have stated that the most lethal industrial factor experienced in developing countries is ON. In Zimbabwe, the Factories and Works Regulations (General) 1976 (Zimbabwe Government, 1996b) specifies that ‘no one shall be subjected to sound levels exceeding the limits, 90 dBA, unless that individual is equipped with ear protectors’. Past empirical studies have shown substantial evidence of positive relationship between workplace noise and blood pressure as well as the heart rate of workers in the steel industry (Zamanian, Rostami, Hasanzadeh, & Hashemi, 2013), speech recognition (Prell & Clavier, 2017), quality of life (Otoghile, Onakoya, & Otoghile, 2018) and hearing abilities of workers in the quarry sector (Gyamfi, Amankwaa, Sekyere, & Boateng, 2016). Therefore, it is important to understand how ON could influence attitudes towards ON exposure and perceived susceptibility to hearing loss (PSTHL).

### Attitudes towards occupational noise exposure

An attitude is a person’s enduring favourable or unfavourable evaluations, emotional feelings and action tendencies towards a certain behaviour (Roberts-Lombard & Parumasur, 2017). Attitudes can be characterised as the general assessment of an individual playing out a particular conduct (Celik & Yilmaz, 2011). If the behavioural attitude is positive, the willingness of the individual to execute that specific behaviour rises (Ajzen, 1991). In light of the above clarifications, it could be seen that if an individual has a horrible frame of mind towards ON, their activity execution (job performance) is probably going to be influenced. Therefore, with the end goal of this investigation, the researchers are seeking to determine attitudes towards ON exposure. Gyamfi et al. (2016) clarified that initial sensitivity to ambient noise is perceived as a rise in the hearing threshold. Nevertheless, Sogebi, Amoran, Iyaniwura and Oyewole (2014) argued that apart from being widely accepted as an inconvenience and a source of attention loss, workplace noise exposure often affects the output of employees. The authors also cautioned that the hearing loss was caused in workers who are regularly exposed to workplace noise. A research conducted by Nyarubeli, Tungu, Bratveit and Moen (2019) has reported that in developing nations, NIHL is an emerging problem of public health. Nyarubeli et al. (2019) credit this to rapid industrialisation as well as failure by institutions, that is, government departments and organisations, to provide preventative measures. For instance, regulators, such as inspectorates from the ministry of labour, International Labour Organisation (ILO), Zimbabwe Office, the Ministry of Public Service, Labour and Social Welfare, and the National Social Security Authority (NSSA), need to accommodate precautionary measures against noise. Rantanen, Lehtinen, Valenti and Lavicoli (2017) acknowledged that there is scant literature and awareness amongst the working population about noise exposure. Nyarubeli et al. (2019) suggest that this shortage of literature has an impact on the information that workers have on ON.

### Perceived susceptibility to hearing loss

Perceived susceptibility, according to Cornford (2018), is the feeling of being vulnerable to a disease and the degree to which the patient feels he or she is at risk of reaching that condition. Safari, Ahmadi, Sare and Ghorbanideh (2018) have stated that because noise is an occupational hazard, being exposed to tremendous ON could result in a range of problems related to one’s work. A research carried out by Khala, Al-Shereda and Al-Ansary (2012) has revealed that, since the industrial revolution, hearing loss because of exposure to workplace noise has been a topic of discussion. According to Ismail et al. (2013), NIHL is a short-term or long-term sensorineural impairment of hearing caused by exposure to ON for a considerable period of time. Khala et al. (2012) have reported that exposure to a loud sound or exposure to a sound for an ongoing duration destroys hair cells in the inner ear, resulting in noise-induced threshold shift (NITS), a disorder attributed solely to noise. Timmins and Granger (2010) conclude that hearing loss caused by noise could occur immediately or slowly. Khala et al. (2012) have further emphasised that the threshold change caused by the intensity and frequency of noise (NITS) may be either temporary or permanent.

### Job performance

Job performance in this study is regarded as the behaviours, attitudes and outcomes in which individual employees engage or bring about that contribute to the goals of an organisation (Mafini, 2015). Job performance is influenced by three main factors (Farh, Seo, & Tesluk, 2012; Kacmar, Harris, Collins, & Judge, 2009), namely, declarative information (reality knowledge, concepts and objects), organisational knowledge and expertise (declarative knowledge execution capability) and motivation (engagement preference, commitment and persistence). Workplace noise was found to be one of the most important predictors of job performance (Al-Omari & Okasheh, 2017). Based on its results, job performance is correlated with a variety of organisational outcomes, including customer service and product quality (Blignaut, 2011), manager–employee relations and employee turnover (Dalal & Hulin, 2008), and job satisfaction (Bono & Judge, 2003). Therefore, in terms of its meaning, job performance appears to be multifaceted and has a wide range of applications in terms of its outcomes.

## Theoretical model and hypotheses formulation

**FIGURE 1:**
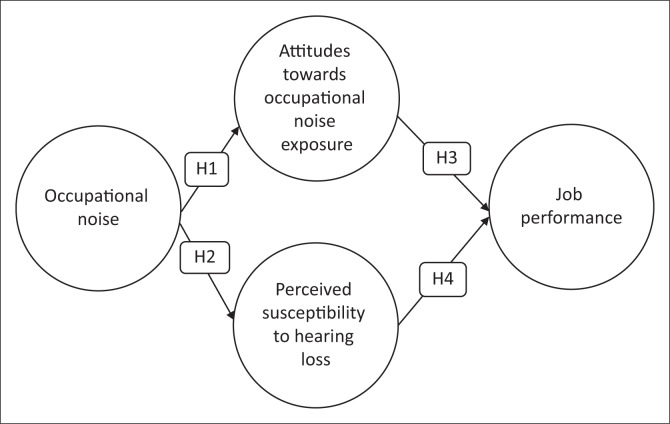
Theoretical model.

A theoretical model for the management of empirical examination is proposed, as shown in Figure 1. The hypothesised relations between research constructs are discussed hereafter.

### Occupational noise and attitudes towards occupational noise exposure

It is important to elucidate on the connection between ON and attitudes towards exposure to ON. A study conducted by Alzahrani et al. (2018) has illustrated that 30% of reported cases of hearing loss are ascribed to exposure to ON. According to Keppler, Dhooge and Vinck (2015), a growing body of literature concurs that excessive ON affects the attitudes of employees towards exposure to occupation noise. In a study carried out by Abulude et al. (2018), ON is taken as a nuisance occupational exposure. On the contrary, in Gunny et al.’s (2018) study, ON is seen as a distressing workplace noise exposure. Therefore, the following hypothesis was formulated:

*H1*: Occupational noise has a positive and a significant impact on attitudes towards ON exposure amongst SME workers.

### Occupational noise and perceived susceptibility to hearing loss

Preliminary work on PSTHL undertaken by Cornford (2018) revealed that exposure to ON results to susceptibility to hearing loss. In addition, a study carried out by Khala et al. (2012), which determined the effect of noise on Basrah Petrochemical factory’s workers, revealed that workplace noise leads to loss of hearing. World Health Organisation (WHO, 2015) further affirms that ON damages the auditory system, thereby resulting in hearing loss. Research carried out by Kurt, McKenna, Gunbeyaz and Turan (2017), which investigated workplace noise exposure in a ship recycling yard, revealed that workers were at increased risk of induced hearing loss as a result of sustained, dangerous levels of noise at work. Hence, a second hypothesis is formulated on the basis of this presumption:

*H2*: Occupational noise has a positive and a significant impact on perceived susceptibility to hearing loss amongst SME workers.

### Attitudes towards occupational noise exposure and job performance

Studies conducted by Klatte, Berstrom and Lachmann (2013) and Hume, Brink, and Basner (2012) have revealed that ON has an impact on job performance. In the same vein, Nassiri et al. (2013) concur that exposure to ON significantly affects the performance of employees. Abbasi et al. (2019) further buttress the finding that ON exposure increases stress levels and job dissatisfaction, which, in turn, affects the overall job performance of employees. Deriving from the above studies and empirical evidence, following is hypothesised:

*H3*: Attitudes towards ON exposure has a positive and a significant impact on job performance of SME workers.

### Perceived susceptibility to hearing loss and job performance

According to Cornford (2018), the perceived susceptibility is the feeling of being susceptible to a disease and the degree to which the patient feels he or she is at risk of developing the condition. A study by Wagner-Hartl and Kallus (2018) shows that hearing loss has a considerable impact on employee’s job performance. Furthermore, A study conducted by Edwards et al. (2011) in South Africa expressed that hearing loss influences the job performances of employees. Hence, it can be conceivably hypothesised that:

*H4*: Perceived susceptibility to hearing loss has a positive and a significant association with the job performance of SME workers.

## Research purpose

Inferring from the hypothetical statements formulated in the preceding section, the purpose of the current research is to determine the impact of ON on attitudes towards ON exposure, susceptibility to hearing loss and job performance of manufacturing SMEs workers in Zimbabwe. This would help devise strategies and establish preventive measures that could help minimise the risk of work hearing loss amongst exposed populations. The specific aims of this work are formulated as follows:

- to determine the impact of ON on ON exposure
- to examine the impact of ON on PSTHL
- to assess the impact of attitudes towards ON exposure on job performance
- to determine the impact of PSTHL on job performance.

### Methodology

The research philosophy of this study was positivism. Hence, the study embraced a quantitative approach. Quantitative research is to be based on a positivist paradigm of measuring variables (Rahman, 2017). Therefore, a quantitative research approach was used for this study. The design was suitable for requesting the information concerning ON, attitudes towards ON exposure, and PSTHL and job performance. The data were collected in 2019, and the population included workers from SMEs located in Magaba, Mbare. Self-administered questionnaires were distributed to respondents from 350 manufacturing SMEs. Of the distributed questionnaires, 250 were completed satisfactorily, resulting in a response rate of 71.4%. The research included both male and female workers of SMEs, who were aged 18 years and more. Nonetheless, lack of reliable and accurate list of participants means the research was amenable to sampling procedures based on non-probability. The convenience sampling technique was used in the absence of an appropriate sampling frame (Churchill, Brown, & Suter, 2010), as it has been cited as very beneficial practical method.

### Measurement instrument and questionnaire design

All the constructs in this article, except respondents’ demographic profile, were measured on a five-point Likert rating scale with the endpoints of 1 = strongly disagree to 5 = strongly agree. The multi-item approach were adopted from past investigations and modified to fit the setting of the present examination (Cornford, 2018; Kaynak, Toklu, Elci, & Toklu, 2016; Nyarubeli et al., 2019; Realyvásquez et al., 2016). The construct and scale sources, scale items utilised and Cronbach’s alpha values for the scales are given in Table 1.

**TABLE 1:** Measurement scales and their sources.

Construct and source	Description	Cronbach’s alpha
*Occupational noise:* As adapted from Realyvásquez et al. (2016)	The tasks are performed in comfortable noise environments. Employees are isolated from machines which emit high levels of noise. In this company, regular measurements of the noise level are carried out.	0.600
Attitudes towards *occupational noise exposure:* As adapted from Nyarubeli et al. (2019)	In my opinion, my employer should compensate me if I have hearing loss from this work ‘am doing. I think, I can use hearing protective devices effectively without any training. I believe, wearing hearing aids during my job is a burden and an inconvenient one. In my opinion, it is not important to have regulations on noise control at my site. I feel, wearing hearing protective devices in high noise levels is not my sole responsibility. I feel that it is our shared responsibility to reduce exposure to noise at the workplace. I feel that my employer should be informed if I have hearing loss. I think that an ear screening programme (audiometry) at my workplace is not so important. I believe, I can consult traditional healer when I have hearing loss. I feel, I should not bother on high noise levels as long as I am energetic and healthy. I think, hearing loss is because of other factors such as age and ear injury and not because of noise exposure. I believe, working in a noisy environment for one≈shift a day does not cause hearing loss. I don’t work at noise levels that could damage my hearing.	0.810
*Perceived susceptibility to hearing loss:* As adapted from Cornford (2018)	My hearing is likely to get worse in the future. I may lose my hearing. I am unlikely to lose my hearing because my family does not experience hearing loss. I have heard, you are expected to check your hearing now and then.	0.621
*Job performance*: As adapted from Kaynak et al. (2016)	I still complete the job description duties at my≈place of work. My duties are fulfilled as required by my work. I have failed to fulfil my core tasks. I am not neglecting the tasks which my job requires. I carry out the formal tasks which my job requires.	0.853

### Data collection

The data were collected in Magaba, Mbare, Zimbabwe. Data collection refers to the detailed and systematic compilation of views and opinions that have the ability to resolve the research issue (Murthy & Bhojanna, 2010). For this analysis, a data collection survey method was suitable because a quantitative approach was used. McDaniel and Roger (2007) clarify that survey approach is used to obtain information about participants, including their views, attitudes and behaviour. Similarly, Blumberg et al. (2008) argue that this approach is the most preferred methodology for collecting essential information. Precisely for data accumulation, the investigation utilised a structured questionnaire comprising a list of questions. The utilisation of the questionnaire was aimed at producing essential data valuable for enhancing response rate during the survey.

### Ethical considerations

Permission was obtained from the administration of the Harare City Council. The researchers acquired the permission letter which permitted them to gather information from manufacturing SME workers. Ethical clearance approval was affirmed genuinely, and this study acted as per the ethical benchmarks of scholastic research, which incorporate, in addition to other things, protecting the identities of respondents and guaranteeing secrecy of accumulated information obtained from respondents.

### Data analysis

Data were analysed using the Social Sciences Statistical Package (version 25.0) for descriptive statistics, whilst model fit and path modelling were carried out using Analysis of Moment Structures (AMOS) statistical software (version 25.0). The next section contains descriptive statistics related to respondents’ profiles.

## Research results

### Demographic profile summary

Table 2 shows the participants’ representation. The respondents were asked to report their demographic data, including gender, age and educational levels. The respondents were mainly males (80.0%). The average age of the respondents was between 40 and 49 years (18.4%). As far as the level of education was concerned, 37.6% (*n* = 94) of respondents had a diploma, 30.0% (*n* = 75) confirmed having a degree, 28.4% (*n* = 71) revealed having some basic training and the remaining 4.0% (*n* = 10) had no formal education.

**TABLE 2:** Scale accuracy analysis.

Research constructs		Mean values	SD values	Item to total correlation values	*α*	CR	AVE	Factor loadings
Codes	Code items							
ON	POS1	3.58	1.012	0.610	0.797	0.800	0.590	0.874
	POS2	3.67	1.085	0.539				0.838
	POS3	4.11	1.016	0.518				0.543
ATONE	ATONE1	3.75	1.055	0.528	0.831	0.947	0.578	0.762
	ATONE2	3.87	1.035	0.619				0.728
	ATONE3	3.79	1.074	0.593				0.732
	ATONE4	3.59	1.077	0.608				0.778
	ATONE5	4.63	1.328	0.737				0.778
	ATONE6	4.45	1.143	0.897				0.771
	ATONE7	4.86	1.424	0.506				0.792
	ATONE8	4.59	1.199	0.801				0.769
	ATONE9	4.51	1.261	0.781				0.704
	ATONE10	4.82	1.168	0.790				0.774
	ATONE11	4.22	1.431	0.731				0.783
	ATONE12	3.92	1.477	0.531				0.755
	ATONE13	3.71	1.593	0.605				0.753
PSTHL	PSTHL1	4.21	0.927	0.530	0.826	0.820	0.530	0.693
	PSTHL2	3.96	1.011	0.543				0.776
	PSTHL3	3.88	1.001	0.561				0.636
	PSTHL4	3.85	0.978	0.598				0.787
JP	JP1	4.12	0.881	0.596	0.931	0.962	0.838	0.972
	JP2	3.86	1.047	0.501				0.972
	JP3	4.08	0.884	0.598				0.632
	JP4	4.46	1.675	0.567				0.981
	JP5	4.87	1.644	0.673				0.969

ON, occupational noise; ATONE, attitudes towards occupational noise exposure; PSTHL, perceived susceptibility to hearing loss; JP, job performance; SD, standard deviation; CR, composite reliability, AVE, average variance extracted.

### Scale accuracy analysis

The scale accuracy analysis is described in Table 2, accompanied with a discussion on the reliability and validity of measurement scales.

### Reliability and validity of measured items

The internal consistency of the measured items was evaluated using the Cronbach’s alpha coefficient, item-to-total correlation values, factor loadings, extracted average variance and composite reliability (CR). According to Hair, Black, Babin and Anderson (2010), the alpha value should be greater than 0.6 for constructs to be considered reliable. The test of reliability (Table 2) for all the constructs suggests alpha values to be greater than 0.6. In addition, item-to-total correlation values ranged from 0.501 to 0.897, which reach the appropriate threshold of 0.5 (Anderson & Gerbing, 1988). This means that measurement instruments were accurate, and convergent validity was present. The loadings should be more than 0.5 (Hair, Black, Babin, Anderson, & Tatham, 2006), as shown in Table 2. In this study, the factor loadings ranged from 0.543 to 0.981, reaching the required value of 0.5.

Composite reliabilities and average variance extracted (AVE) for each construct were computed by using the following formulae proposed by Fornell and Lacker (1981, p. 22):

$${\text{CR}}_{\eta }={\left(\Sigma \lambda yi\right)}^{2}/\left[{\left(\Sigma \lambda yi\right)}^{2}+(\Sigma \varepsilon i)\right],$$ [Eqn 1]

where CR*η* is the composite reliability, (Σ*λyi*)^2^ is the square of the summation of factor loadings and (Σ*εi*) is the summation of error variances.

$${\text{V}}_{\eta }=\Sigma \lambda yi^{2}/(\Sigma \lambda yi_{2}+\Sigma \varepsilon i),$$ [Eqn 2]

where V*η* is the AVE, Σ*λyi*^2^ is the summation of the square of factor loadings and Σ*εi* is the summation of error variances.

As shown in Table 3, the minimum CR value of 0.80 is well above the recommended value of 0.6 (Hulland, 1999), whilst the lowest AVE value of 0.53 is also above the recommended value of 0.4 (Fraering & Minor, 2006). It demonstrates the achievement of convergent validity, and shows the excellent internal consistency and reliability of the measuring instruments used. As such, all constructs showed a sufficient degree of discriminating validity (see Table 3). Such findings have, by and large, provided evidence of acceptable levels of reliability of the study scale (Chinomona & Chinomona, 2013, p. 20; Chinomona & Mofokeng, 2016).

**TABLE 3:** Sample demographic characteristics.

Characteristics	Frequency	Percentage
**Gender**		
Male	200	80.0
Female	50	20.0
**Age distribution of respondents (years)**		
18–30	31	12.4
31–39	75	30.0
40–49	46	18.4
50–59	81	32.4
60 years and above	17	6.8
**Level of education**		
No formal education	10	4.0
Basic education	71	28.4
Diploma	94	37.6
Degree	75	30.0
**Total**	**250**	**100.0**

The discriminant validity refers to items measuring different concepts (Field, 2013). The findings of the discriminant validity study are reported in Table 4. As depicted in the table, all correlation coefficients of this study were below 0.70, thereby confirming the theoretical uniqueness of each variable in this research (Field, 2013).

**TABLE 4:** Inter-correlations for independent and dependent variables.

Variables	ON	ATONE	PSTHL	JP
**ON**	**1**	-	-	-
**ATONE**	0.431*	**1**	-	-
**PSTHL**	0.423*	0.269*	**1**	-
**JP**	0.249*	0.534*	0.425*	**1**

ON, occupational noise; ATONE, attitudes towards occupational noise exposure; PSTHL, perceived susceptibility to hearing loss; JP, job performance.

* , Correlation is significant at 0.01 level (2-tailed).

### Structural equation modelling approach

A double-step approach was followed to conduct SEM. Firstly, the psychometric properties of the measurement model were inspected through confirmatory factor analysis, whilst the second step (SEM) concentrated on testing the structural model and determining causal relationships amongst variables. The following section provides the model fit investigation of these two stages.

### Model fit analysis

Acceptable model fit indices used in this study included the following: chi-square/degree of freedom (*χ*^2^/(*df*)) ≤ 3.00, comparative fit index (CFI) ≥ 0.90, Tucker and Lewis index (TLI) ≥ 0.90, incremental fit index (IFI) ≥ 0.90, normative fit index (NFI) ≥ 0.90, goodness of fit (GFI) ≥ 0.90 and the root mean square error of approximation (RMSEA) ≤ 0.08 (Lysons & Farrington, 2012). Table 5 provides the general model fit indices for both confirmatory factor analysis (CFA) model and SEM.

**TABLE 5:** General model fit statistics.

Fit indices	Acceptable fit indices	CFA (measurement model)	SEM (structural model)
Chi-square/degree of freedom (*df*)	< 3.0	1.529	1.636
Incremental fit index (IFI)	> 0.90	0.942	0.941
Tucker-Lewis index (TLI)	> 0.90	0.962	0.938
Comparative fit index (CFI)	> 0.90	0.947	0.938
Normative fit index (NFI)	> 0.90	0.945	0.934
Goodness of fit (GFI)	> 0.90	0.934	0.921
Root mean square error of approximation (RMSEA)	< 0.08	0.053	0.044

CFA, confirmatory factor analysis; SEM, structural equation modelling.

### Outcome of hypotheses testing

For the present study, hypothesis testing was conducted by assessing path coefficient values and *p*-values for structural model (Table 6). Path coefficients were generated in the model by the causal relationships proposed in this analysis. Based on the coefficients, the hypotheses were examined.

**TABLE 6:** Summary of hypotheses testing.

Relationships	Hypothesis	Path coefficient *Β*	*p*	Remarks
**ATONE ← O N**	**H1**	0.364	***	Supported
**PSTHL ← ON**	**H2**	0.257	***	Supported
**JP ← ATONE**	**H3**	0.232	***	Supported
**JP ← PSTHL**	**H4**	0.573	***	Supported

ON, occupational noise; ATONE, attitudes towards occupational noise exposure; PSTHL, perceived susceptibility to hearing loss; JP, job performance.

*** , significance level *p* < 0.01.

### Outcome of testing hypothesis 1

Hypothesis 1 states that ‘occupational noise has a positive and significant impact on attitudes towards occupational noise exposure amongst SME workers’. In light of the outcomes of the last model tested, the connection between ON and attitudes towards ON exposure shows coefficient *β* = 0.364 at *p* < 0.01. This shows that H1 is supported. Subsequently, it is noticed that ON impacts the attitudes towards ON exposure of workers within SMEs. It is likewise fundamental to make reference to the fact that these discoveries authenticate the outcome of the research conducted by Ranga, Yadav, Yadav, Yadav, and Ranga (2014, p. 117), who explained that ON is a key worry for the workers utilised in the work environment having tenacious exposure to noisy atmosphere. The consequences of this examination, in addition, discover support in the investigation conducted by Mohammadi et al. (2016, p. 1740), who exhibited that ON is amongst the most genuine business-related dangers, which, notwithstanding ON exposure, can deliver troublesome impacts on worker’s physical and mental prosperity.

### Outcome of testing hypothesis 2

**FIGURE 2:**
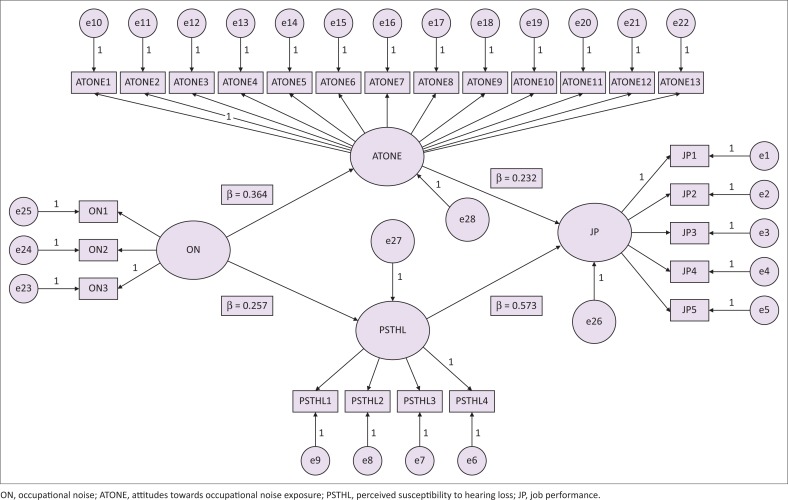
The final structural model of the study.

Hypothesis 2 affirms that ‘occupational noise has a positive and a significant impact on perceived susceptibility to hearing loss amongst SME workers’. The structural model (Figure 2) displays connection between ON and PSTHL as evidenced by *β* = 0.257 at *p* < 0.01. Subsequently, H2 is supported. The outcomes imply that ON impacts apparent vulnerability to hearing loss amongst labourers in assembling SMEs. The outcomes received from testing this speculation concur with literature. For example, Cornford (2018) uncovered that presentation to ON brings about powerlessness to hearing loss. Lie et al. (2016) underscored the positive and significant impact of workplace noise on perceived vulnerability to hearing loss amongst manufacturing SME workers. Moreover, a study conducted by Stucken and Hong (2014) complements that ON may add to the present moment or long haul edge changes; in any case, even momentary limit changes may incite a worker to conceivable enduring hearing loss.

### Outcome of testing hypothesis 3

Hypothesis 3 expresses that ‘attitudes towards occupational noise exposure has a positive and significant impact on job performance of SME workers’. In view of the results of the path model, the relation between attitudes towards ON exposure and job performance shows *β* = 0.232 at *p* < 0.01. This proof exhibits that H3 is bolstered. The outcomes suggest that attitudes towards ON exposure impact job performance of manufacturing SME workers. This is in accordance with the findings of Hume et al. (2012) that attitudes towards ON affect job performance. They are also in accordance with the findings of Nassiri et al. (2013), who examined the impact of clamour on human execution. Their investigation uncovered that exposure to ON was found to be basic in diminishing job performance. It was additionally found by Chandrasekar (2011, p. 1) that ON presentation has a quick and imperative connection with efficiency and job performance of workers.

### Outcome of testing hypothesis 4

Hypothesis 4 expresses that ‘perceived susceptibility to hearing loss has a positive and a significant association with the job performance of SME workers’. In light of the results during the hypothesis testing stage, the relation between PSTHL and job performance shows *β* = 0.573 at *p* < 0.01. This proof exhibits that H4 is supported. The outcomes suggest that PSTHL has an association with job performance of manufacturing SME workers. It is additionally worth referencing that these discoveries fortify the outcomes arrived at in the investigation carried out by Guarnaccia, Mastorakis and Quartieri (2013, p. 38), who set up that PSTHL influences job performance of workers. Hong, Kerr, Poling and Dhar (2013) confirm that there are various extreme negative results of hearing loss, including correspondence hindrance that could liberally affect down-to-earth limit, worker certainty and job performance.

### Managerial implications

The current examination involves a few implications for scholastics. A study of the findings indicates that ON and PSTHL affect each other, as attested by a path coefficient of 0.257. For scholastics in the field of occupational hearing loss and independent company board, this discovery improves their comprehension of the connection between ON and PSTHL, making this investigation a significant addition to existing literature.

On the practitioners’ side, the outcomes of the present investigation provide arrangements from which SME managers and supervisors can profit. Given the powerful connection between helplessness to hearing loss and job performance, as demonstrated by path coefficient of 0.573, directors of emerging SMEs in Magaba, Zimbabwe, should focus on methodologies that limit ON inside their workplace, as noise is a contributing variable to hearing loss.

### Conclusions, limitations and future research directions

The present investigation affirms that ON has a positive and significant impact on attitudes towards ON exposure and PSTHL. Moreover, attitudes towards ON exposure and PSTHL had a positive and significant effect on job performance. Regardless of the pertinent bits of knowledge offered by this study, current discoveries ought to be deciphered, considering significant methodological shortcomings. Firstly, information utilised has been collected from manufacturing SME workers and not those in supervisory positions. The current authors concede that the outcomes would have been more thorough and intensive if information from both cadres was obtained. Furthermore, the present investigation was restricted to a small manufacturing SMEs in one territory of Zimbabwe, and surveys were utilised to gather information from respondents. This leads to the issue of generalisation of findings to the bigger populace, and the degree to which the consequences of the investigation might be summed up in different settings and situations. Thirdly, the investigation technique embraced was quantitative. A qualitative enquiry could have incited knowledgeable and more beneficial data, for example, acquiring views on workplace noise from focus groups of SME workers and reporting the same as verbatim responses. Along these lines, a subjective procedure could have uncovered that had it formed part of the methodological strategy. Eventually, utilising triangulation would eradicate bias of the common method used. The future investigations could take into account these methodological shortcomings. Besides, similarities and differences between the findings of this study and those acquired from different firms in various areas or through meta-investigations could likewise be considered later on. Moreover, it is imperative to note that lack of noise and audiometry outcomes is also a major limitation of this study; even if a retrospective outcome of previous surveys and medical surveillance by workers had been furnished, this would have given a better picture of the problem under investigation.
